# Opening the black box of selection

**DOI:** 10.1007/s10459-019-09925-1

**Published:** 2019-10-09

**Authors:** Sanne Schreurs, Kitty Cleutjens, Carlos F. Collares, Jennifer Cleland, Mirjam G. A. oude Egbrink

**Affiliations:** 1 Department of Educational Development and Research, School of Health Professions Education (SHE), Faculty of Health, Medicine and Life Sciences, Maastricht University, P.O. Box 616, 6200 MD Maastricht, The Netherlands; 2 Department of Pathology, Faculty of Health, Medicine and Life Sciences, Maastricht University, Maastricht, The Netherlands; 3 Centre for Healthcare Education Research and Innovation (CHERI), University of Aberdeen, Foresterhill, Aberdeen, UK; 4 Department of Physiology, School of Health Professions Education (SHE), Institute for Education, Faculty of Health, Medicine and Life Sciences, Maastricht University, Maastricht, The Netherlands

**Keywords:** Selection, Medical school, Admissions, Validity, Construct validity

## Abstract

Medical school selection is currently in the paradoxical situation in which selection tools may predict study outcomes, but which constructs are actually doing the predicting is unknown (the ‘black box of selection’). Therefore, our research focused on those constructs, answering the question: do the internal structures of the tests in an outcome-based selection procedure reflect the content that was intended to be measured? Downing’s validity framework was applied to organize evidence for construct validity, focusing on evidence related to content and internal structure. The applied selection procedure was a multi-tool, CanMEDS-based procedure comprised of a video-based situational judgement test (focused on (inter)personal competencies), and a written aptitude test (reflecting a broader array of CanMEDS competencies). First, we examined content-related evidence pertaining to the creation and application of the competency-based selection blueprint and found that the set-up of the selection procedure was a robust, transparent and replicable process. Second, the internal structure of the selection tests was investigated by connecting applicants’ performance on the selection tests to the predetermined blueprint using cognitive diagnostic modeling. The data indicate 89% overlap between the expected and measured constructs. Our results support the notion that the focus placed on creating the right content and following a competency-blueprint was effective in terms of internal structure: most items measured what they were intended to measure. This way of linking a predetermined blueprint to the applicants’ results sheds light into the ‘black box of selection’ and can be used to support the construct validity of selection procedures.

## Introduction

The purpose of medical school selection is to recruit students who will perform well at medical school as well as in their future career as a doctor (Bandiera et al. 2015). To achieve this, many selection procedures are now outcome-based (e.g. Frohlich et al. 2017; Patterson et al. 2018; Prideaux et al. 2011; Schreurs et al. 2018; Terregino et al. 2015): ‘beginning with the end in mind’. To this purpose, the cognitive and (inter)personal competencies or qualities needed throughout the study program and in future work are integrated as constructs into the selection process (Cleland et al. 2012; Patterson et al. 2016). Selection procedures typically consist of multiple tools, with each university individually choosing and combining constructs (i.e. competencies or qualities) and tools (Cleland et al. 2012; Patterson et al. 2016; Schreurs et al. 2018), often without proper justification. Up to now, most research has focused on the utility of individual selection tools, showing for example that unstructured interviews are neither reliable nor valid, while Multiple Mini Interviews (MMIs) show better psychometric qualities; that previous academic attainment predicts later academic attainment; that there is a plethora of written tests whose psychometric qualities vary with each variation in format and construct; and that the situational judgment test (SJT) may be a useful tool in medical school selection (e.g. Cleland et al. 2012; Patterson et al. 2016, 2018; Prideaux et al. 2011).

Because selection research has typically focused on the qualities of one particular tool or method in its own right, only few studies have looked at combinations of tools as applied by many medical schools. Studies investigating combined tools have typically focused on incremental validity: whether one tool has predictive value above and beyond another tool (McManus et al. 2013; Patterson et al. 2016; Schreurs et al. 2018; Tiffin et al. 2016). Moreover, to date, there has been no systematic consideration of which constructs (e.g. collaboration or empathy) are actually assessed in medical school selection procedures, and whether this is in line with what was intended from their outcome-based focus (Christian et al. 2010; Wilkinson and Wilkinson 2016). This means that selection may be considered a sort of ‘black box’ (Kreiter 2017; Kulasegaram 2017; Lievens et al. 2008), a paradoxical situation in which selection tools may predict outcomes but which constructs are actually doing the predicting is uncertain (Cleland et al. 2014; Crossingham et al. 2011; Tiller et al. 2013). It is essential to know more about what is *actually* being measured (i.e. the construct validity of selection; e.g. Christian et al. 2010; Hecker and Norman 2017; Kreiter 2017; Kulasegaram 2017; Patterson et al. 2017) in order to determine whether the intended constructs are measured. Research on this subject is sorely missing (Hecker and Norman 2017; Kulasegaram 2017), and would not only greatly benefit the defensibility of selection procedures (Kreiter 2017), but would also be a first step in the direction of creating more theory-based selection procedures (Patterson et al. 2018; Prideaux et al. 2011). Moreover, conducting studies on construct validity yields practical implications for selection. For example, if the intended constructs are not measured and the predictive value is insufficient, the selection committee should go back to the drawing board, since the procedure is neither effective nor defensible or fair (Patterson and Zibarras 2018). Alternatively, if there is predictive value but the intended constructs are not measured, where the predictive value is coming from should be investigated in order to avoid ‘being reliably wrong’ (Patterson and Ferguson 2012) and measuring an unrelated construct that, by chance, correlates with study success (e.g. shoe-tying-skills could be predictive of medical school performance, but cannot defensibly be used as a selection tool). All in all, research on construct validity can help the field of selection for medicine move forward in terms of theory and practice.

One way to systematically assess whether we are measuring the constructs we want to measure, and to investigate possibilities for improving validity, is by applying a validity framework. Validity frameworks provide guidelines on how and what information to gather on assessment methods [in this case the medical school selection process, given selection can be considered the first assessment in medicine (Cleland et al. 2012)] to investigate whether an assessment is applicable for the proposed use. These frameworks also stimulate researchers to view their assessment from different perspectives and take various sources of information into account. Examples of the frameworks that are used within the field of medical education are Kane (1992; also see Cook et al. 2015), Messick (1995), and Downing and the Standards for educational and psychological testing (AERA, APA and NCME, 2014; Downing 2003). Each of these frameworks overlap to a certain degree. Downing explicitly intended his framework to inform research on assessment within medical education, and his framework and the closely related ‘Standards’ have been used in several studies within (Kelly and O’Flynn 2017; Mink et al. 2018) and outside (Sorrel et al. 2016) of medical education.

The ultimate aim of the current study was to address the gap in knowledge with respect to the construct validity of medical school selection procedures. This was done by focusing on the content of the procedure on the one hand, and the internal structure of the procedure on the other. The specific question to be answered in the current study was: do the internal structures of the tests in the second round of the selection procedure reflect the content that was intended to be measured? We selected Downing’s framework as the means to organize the evidence for construct validity of the tools in the second round of a multi-tool, outcome-based selection procedure (more explanation on the selection procedure itself is provided in the methods section).

## Methods

### Context

This study was performed at Maastricht University Medical School (MUMS) in the Netherlands. MUMS administers a multi-tool, outcome-based selection procedure. The selection procedure consists of two rounds containing three tools (hence, multi-tool). In the first stage, applicants complete a pre-structured online portfolio, focusing on previous academic attainment, extracurricular (distinguishing) abilities, and their fit with problem-based learning and the MUMS medical curriculum. This first stage is used as a broad-brush pre-screening to limit the amount of applicants that proceed to the second part of the procedure. The second stage, a selection day at MUMS, consists of a Video-based Situational Judgment Test (V-SJT) and a Written Aptitude Test (WAT), both of which contain items aimed at measuring predetermined competencies (see below Schreurs et al. 2018). In this second stage, a more fine-grained selection takes place. The current study focused on the second stage, and therewith on two tools within the selection procedure at MUMS: the V-SJT and the WAT.

The MUMS selection procedure is outcome-based, as it is based on a blueprint of competencies derived from the CanMEDS framework, a well-known and internationally accepted outcome framework for medical school (Frank 2005; van Herwaarden et al. 2009). The CanMEDS describe seven roles: Medical Expert, Communicator, Collaborator, Organizer (Leader in the 2015 edition), Health Advocate, Scholar and Professional. In the second round of the selection procedure, the V-SJT focuses on the (inter)personal competencies in the CanMEDS, while the WAT more broadly assesses aptitude for (inter)personal as well as more cognitively loaded CanMEDS roles. The applicants’ results on both tests were converted into z-scores, averaged per test, and the means of the two tool-averages were used to create the rank order of applicants, on which they were selected or rejected.

The MUMS selection procedure as a whole has been studied previously for its predictive value and cost-effectiveness (Schreurs et al. 2018a, b). As stated above, the current study focused on what is actually measured during the second stage (i.e. V-SJT and WAT) of the selection procedure. To this purpose, data from all 547 candidates in the second round of the 2016 selection procedure were investigated.

### Ethical approval

Applicants were asked to give their informed consent for the use of their selection and assessment data for research purposes. It was made clear that not taking part in the study would not adversely influence their progression. Participant data were anonymized before they were shared with the research team. The study was approved by the Ethical Review Board of the Netherlands Association for Medical Education (NVMO; file number 2018.8.5).

### Validity framework

We consulted the literature on contemporary validity frameworks in order to assess the validity of the selection procedure. As stated before, we chose Downing’s framework to organize the validity evidence in the current study, since it is applicable to assessment systems such as a selection procedure. In brief, Downing (2003) defines five sources of evidence for construct validity: content (evidence supporting the content of the assessment, such as the thorough development of its blueprint), response processes (evidence showing that the test-takers do in fact employ the processes that were intended to be employed, for example as measured by eye-tracking or trace data), internal structure (evidence related to the structure of the test, for example item quality and factorial structure), relationship to other variables (evidence relating the performance on the test to performance on another test, which should have the expected relationship), and consequences (evidence related to the impact the score on the test has on the test-taker and in how far these are intended and positive/negative). For more information on the framework, see Downing (2003). The research question set forth for the current study was answered by focusing on two of these sources of evidence: content and internal structure. Content evidence pertains to the competency-based blueprint used to develop the selection procedure, while internal structure evidence relates to the extent to which the blueprint is reflected in the applicants’ results. Details on the approaches taken to investigate these two sources of evidence are below.

### Content

A qualitative approach based on document analysis, attending the selection committee meetings and checking and confirming the results with the head of the selection committee was used to establish an evidence-base for validity concerning the content of the selection procedure. Document analysis was used to understand the manner in which the blueprint for the selection was established. Furthermore, the head of the selection committee provided additional information on this process, while the lead author of this study attended the selection committee meetings in which the content of the procedure was discussed.

Information was gathered on the development of the blueprint used to design the procedure, the subject matter experts (SMEs) who are members of the selection committee and, hence, in charge of the creation and employment of the blueprint, the relationships between constructs and items, and the representativeness of the items for applicants. The SMEs created the items and paid specific attention to the representativeness of the items for the construct. After reaching consensus on the content and questions in the selection items, the SMEs wrote answer keys to each question: possible answers to those questions and how many points those answer options would result in. If applicants provided an unexpected answer, this was related to the answer key and in doubt, such an answer was discussed in a committee meeting. In addition, to determine the representativeness of the items for applicants, a post-selection questionnaire was employed to investigate whether the applicants found the items representative for what they thought should be assessed in a selection procedure. Participants in the second round of the selection procedure received the post-selection questionnaire after finishing the V-SJT and WAT. It contained 31 questions related to the organization of the selection day, the information that had been provided beforehand, whether the applicants thought the items in the tests were relevant for future medical students and doctors and whether the assessment was complete (i.e. whether they thought there were questions left unasked that would have been important to include in the selection procedure). Since the focus of the current study was on the selection procedure’s representativeness of the items, the reactions to the following statements were taken into account: (1) “The assignments offered me the possibility to present an accurate portrayal of my abilities” (for the V-SJT and the WAT separately), and (2) “In my opinion, the selection procedure as a whole encompasses all aspects needed for the identification of the best suited candidates for the Bachelor of Medicine” (one overarching statement for the entire procedure). Reactions to these statements could be provided on Likert scales of 1 through 5 (1: completely disagree, 2: disagree 3: neutral, 4: agree, and 5: totally agree).

### Internal structure

To assess internal structure, data were gathered on the applicants’ performance on the V-SJT and WAT. The applicants were first graded according to the answer keys determined by the SMEs. In order to enable comparison of the performances on items for the present study, the raw scores were transformed into z-scores per item (a standardized score taking into account the performance of all other applicants with a mean of zero and a standard deviation of one).

Related to internal structure, internal consistency is a very important characteristic. However, Cronbach’s alpha has been criticized for using a tau-equivalent approach to estimate reliability (Peters 2014) and its inability to cope with multi-dimensionality (Sorrel et al. 2016). Since the data used in the current study are multidimensional in two ways (i.e. there are multiple constructs being measured by different items within each test [multidimensionality *between* items] and the items themselves are measuring multiple constructs at the same time, in different compositions per questions [multidimensionality *within* items]), Cronbach’s alpha was considered to be inadequate. Furthermore, the Omega coefficient can account for the multidimensionality *between* items, but not *within* items (Dagnall et al. 2018). Thus, in this study, the internal consistency could not be based on Cronbach’s alpha or the Omega coefficient. An analysis method that is in fact capable of dealing with multidimensionality between as well as within items is the G-DINA, an analysis in the family of cognitive diagnostic models (CDM). The newest version of G-DINA provides a test level accuracy and attribute level accuracy; these results are provided later. A more detailed description of CDM and G-DINA is given below.

Inter-rater and intra-rater reliability was calculated from historical and current data from round two of the MUMS selection procedure. When the selection procedure was being set up in 2011, both types of reliability were assessed formally. Inter-rater reliability was established by having multiple assessors score the same question for a multitude of applicants, and calculating the correlation between these assessments. Intra-rater reliability was established by having single assessors score all applicants on one question, and then having them go back to the answers 2 weeks after they had scored them first, and establishing the correlation between the scores the first and second time these applicants were scored.

An initial exploration of the properties of the items was done using descriptive statistics (i.e. means, standard deviations, item-total correlations) and Cognitive Diagnostic Modeling (CDM, see later), in order to gather information about the overall functioning and fairness of the items. The second-round items were initially analyzed for the group as a whole and later also for subgroups for which no differences were expected (gender, age), in order to investigate possible differential item functioning (DIF). The effect of pre-university grade point average (pu-GPA), which may affect performance, was also investigated. DIF for gender, age and pu-GPA was conducted using independent samples *t*-tests and linear regression analyses. The critical *p* value was set at 0.05. For these analyses, no correction for multiple comparisons was applied as the goal was not to find statistical significance, but to check whether there was possible DIF, i.e. whether there were differences of educational significance (e.g. if gender would determine all scores and therewith who gets selected, this should be changed immediately). However, because of the possibility of finding significant differences by chance, the results should be considered critically.

Next, to answer the main research question, i.e. to identify whether the constructs set forth in the selection blueprint are in fact the constructs measured in the selection process, data on the applicants’ performance were linked with blueprint data. Classical test theory is commonly used for this task (e.g. Kiessling et al. 2016; Lievens et al. 2008; Patterson et al. 2012). However, as stated before, because of the multidimensionality between as well as within items, inherent to selection tools, classical test theory (e.g. Cronbach’s alpha) is inadequate (Sorrel et al. 2016). To overcome this issue, we applied an alternative test theory, cognitive diagnostic modeling (CDM), as this test theory (to the best of our knowledge) is the only one capable of coping with multidimensionality between as well as within items. CDM is comprised of a family of multidimensional categorical-latent trait models that allow the use of latent variables for assessment tools that contain items that measure more than one dimension concurrently (Garcia et al. 2014). In other words, CDM is capable of finding latent variables when there is multidimensionality in the data, both between and within items. CDM is related to Confirmatory Factor Analysis: the structure is provided, and CDM looks at whether that structure is indeed found in the data, or whether alterations to the structure make more sense on the basis of the data provided. Thus, CDM is a confirmatory technique which requires a pre-specified blueprint.

CDM requires two independent sources of input. The first is a so-called Q-matrix, i.e. the abovementioned blueprint, which tells the model which competencies were planned to be assessed in which items. This Q-matrix is tested for accuracy and alterations are proposed; only the constructs already in the blueprint can be ‘found’ by the analysis (i.e. the analysis does not search for additional constructs). The second source is the data on applicant performance. Applicant performance is supplied per item, meaning that even if there were four constructs being measured in one item, there was only one score for that item. It is up to the CDM to disentangle the performances in different items measuring different constructs. Importantly, the applicant performance data must be either binary or ordinal.

In summary, in CDM, the expected structure of the latent variables is provided to the CDM, and this structure is tested. Suppose we have a relatively simple test consisting of ten questions measuring three competencies, X, Y and Z. If a test-taker scores relatively high on items in which we intended to measure X and Y but low on items measuring Z, the model can deduce how this test-taker will score on each specific item based on the results on all items. We can assume that the level of competency in the test-taker does not change during the test; hence, if there is an item that is supposed to measure X and Y, but the test-taker scores low, the item may not measure what it is intended to measure. If at the same time another test-taker scores high, although that second test-taker usually only scores high on Z, the model will propose that this specific item may not be measuring X or Y, but instead is measuring Z. The CDM also creates a file with a grid containing all test-takers and constructs, in which it determines which test-takers are capable in which constructs. For more information on CDM, practical guides to usage and syntax, the reader is referred to George (2015, 2016) or Ravand and Robitzsch (2015); for examples of the use of CDM the reader is referred to Garcia et al. (2014) or Sorrel et al. (2016).

The specific model from the CDM family used in this study is the G-DINA model, a generalization of the “deterministic inputs, noisy and gate” (DINA) model (Ravand and Robitzsch 2015). In G-DINA, each combination of latent variables is called a latent group, which represents one reduced attribute vector and has its own associated probability of success. This allows the G-DINA to paint a more realistic picture of the proportion of variance accounted for each dimension in relation to the original DINA model. This model has been used successfully in competency-based SJTs in areas other than medical education (Garcia et al. 2014). In the current study, a saturated G-DINA model was applied to the V-SJT as well as the WAT. These analyses were conducted separately because the tests are independent from each other. This choice was further supported using model fit indices: AIC (Akaike Information Criterion) and BIC (Bayesian Information Criterion) model fits. The sample size in the current study (n = 547) was too low for more specific models (de la Torre and Lee 2013) or for analysis of more than two levels (i.e. ordinal data) within the G-DINA; over a thousand test-takers would have been needed for either. Therefore, the applicants’ performance in our procedure was converted from z-scores per item (which is how the scores were handled in practice) to 0/1 scores (negative z-scores become 0 and positive z-scores become 1). To our knowledge, this is the first study on medical school selection applying CDM to obtain validity evidence.

The statistical packages used to analyze the descriptive statistics and Differential Item Functioning was SPSS version 24 for Windows (IBM statistics). The package used for the CDM analyses was the programming environment R (www.r-project.org), specifically the G-DINA package (Ma and De La Torre 2017; version 2.4.0).

## Results

### Content

The team of SMEs (n = 8) that formed the selection committee consisted of experts in education and medicine. Most SMEs had multiple roles, including university teacher, medical doctor (hospital or general practice), psychologist, educationalist, study advisor and/or researcher. Furthermore, one bachelor (i.e. pre-clinical) student representative was part of the selection committee. Together, the selection committee comprised all expertise considered necessary, enabling them to create a holistic selection procedure assessing all important competencies. Among the SMEs were also experts on assessment item creation. The team of SMEs was responsible for the representativeness of items and construct-coverage. SME decisions were made during the selection committee meetings on the basis of (discussion until) complete agreement between the members.

The internationally-recognized competency framework CanMEDS (Canadian Medical Education Directives for Specialists; Frank 2005) and its Dutch derivative (van Herwaarden et al. 2009) were used to define the blueprint of the selection procedure. These outcome frameworks describe the end terms of medical school, a level the applicants have not yet achieved. Therefore, the team of SMEs translated the CanMEDS-competencies into so-called derived competencies applicants may already possess at bachelor entry-level, and which can be measured in a selection procedure. The translation took place by first thoroughly inspecting the CanMEDS competencies. Several meetings were used to gather clinical and medical-school related situations representative for these competencies to inform the content of the blueprint (Motowidlo et al. 2016; Patterson et al. 2008, 2010) and group them into clusters, until unanimous agreement was reached within the group of SMEs. These clusters then formed the basis of the derived competencies. In an iterative process, the selection committee discussed these derived competencies, how they should be defined and to what extent they should be measured. The resulting derived competencies were Transfer (i.e. knowledge and information integration), Textual skills, Reasoning, Communication, Collaboration, Organization, Medical and Societal Consciousness, Ethical awareness, Empathy and Reflection. Importantly, these derived competencies always remain central to discussions within the committee while creating assignments for the selection procedure, consciously mapping the assignments to the blueprint. The derived competency Communication, defined as “related to effectively conveying a message, either in a spoken or written manner”, is measured by all open-ended questions in the selection procedure and was, therefore, not included as a separate, distinguishable competency in the current study. The derived competencies, definitions and explanations are summarized in Table 1. The goal of the MUMS selection procedure was to measure these derived competencies.

**Table 1:** Translation of the CanMEDS competencies into a blueprint of derived competencies for the selection procedure

CanMEDS	Derived competencies	Definition	Relation to items/example	Items
Medical expert and scholar*	Transfer^a^	Integrating prior knowledge with new information	Text provides new information on a medical subject, must be combined with secondary school knowledge to find an answer	3
	Textual skills and	Textual comprehension and structuring skills	Reading comprehension; structuring given information into charts/models	3
	Reasoning	Verbal and inductive reasoning (fluid intelligence)	Task like Raven’s matrices (Engle et al. 1999)	
Communicator	Overall communication^b^	Skills related to effectively conveying a message, either in a spoken or written manner	Related to all items, as each item required narrative, written answers: having students actively express themselves towards the assessors	19
Collaborator	Collaboration	Interpreting and responding to (non)verbal communication of others	Related to shared decision-making (collaboration with patients) or PBL small-group sessions (collaboration with peers)	3
Manager	Organization	Planning and time-management skills	An organizational task is presented, students write down which steps to take and how to prioritize; time pressure is induced by length of the test	1
Health advocate	Medical and Societal Consciousness	Awareness of profound developments and whether they can view these from multiple angles	Items concern manners in which to increase well-being, such as advising patients; or communicating given developments to family members	10
Professional	Ethical awareness	Ability to think about and choose a course of action, and provide rationales	Dilemmas are provided, applicants are asked to choose a side, and provide arguments underpinning this choice	9
	Empathy	Degree to which applicants are able to put themselves in someone else’s shoes	Confronted with poignant situations (e.g. terminally ill patient) and asked to expand upon how the patients and loved ones are feeling and coping	7
	Reflection	Ability to think about and consider (own) actions and skills	Applicants are asked to remember the last time they received feedback and reflect on how they responded and what they did with the feedback	12

*Combination of two CanMEDS competencies

^a^knowledge and information integration; related to applying knowledge as in the role of medical expert and “creation, dissemination, application and translation of medical knowledge” as in the role of scholar (Frank 2005)

^b^Including strength of written arguments

Next, tools capable of assessing aptitude for the derived competencies were sought, leading to the application of a V-SJT based on the CASPer (Computer-based Assessment for Sampling Personal characteristics, using short video fragments; Dore et al. 2017). The content of the V-SJT was adapted to the Dutch context, befitting the problem-based learning applied at MUMS. In contrast to the original CASPer, the V-SJT applied in the MUMS selection procedure applied an open-ended format (which is why the SMEs created answer keys before checking the applicants’ answers). In addition, a written aptitude test (WAT) was developed, which follows the V-SJT format as closely as possible (i.e. open-ended, semi-structured questions relating to real-life situations). The number of items in which a construct (i.e. derived competency) was assessed is also shown in Table 1. Importantly, all-but-one constructs were assessed in multiple items, and the vast majority of items assessed multiple constructs; the combination of constructs measured per item varied. By doing so, the SME team ascertained the representation of the derived competencies in the assignments they generated. Total test duration and the number of items needed to achieve a reliable picture was based on assessment literature and CASPer and MMI experiences, indicating how many items/stations are needed to achieve a reliable picture (Dore et al. 2017; Knorr and Hissbach 2014; Thomson et al. 2014; van der Vleuten and Schuwirth 2005): 90 min for 11 items in the V-SJT and 75 min for 8 items in the WAT.

At the end of the selection day, several students stated that there was a lot of time pressure, and that they could not finish all assessments. Therefore, this effect was assessed and included in our analyses (see later). The time pressure effect was more apparent for the V-SJT than for the WAT, because the V-SJT can only be filled in assignment by assignment; there is no skipping or returning to a question: applicants have to answer the question as they see it. The WAT is a paper test that applicants can browse through, possibly decreasing the effect of time pressure. After the selection process, 458 of the applicants answered the questions in the post-selection questionnaire related to the representativeness of items in the procedure positively. The mean score on the statement whether the V-SJT offered the possibility to present an accurate portrayal of abilities was 3.9 [SD 1.0; 95% CI (3.80; 4.00)] on a five-point Likert scale; for the WAT this result was 3.4 [SD 0.9; 95% CI (3.31; 3.48)]. The overarching statement on whether the selection procedure encompassed all aspects needed for the identification of the best suited candidates scored 3.6 [SD 0.9; 95% CI (3.52; 3.68)].

### Internal structure

The test level accuracy (i.e. in how far the test as a whole, including all items, categorizes each applicant into the right category of either possessing the competencies or not) given by the saturated G-DINA analysis was 0.72 for the V-SJT and 0.20 for the WAT. This means that the accuracy was moderate (i.e. between 0.7 and 0.9) for the V-SJT (Swets 1988), but very low for the WAT. However, the data on the attribute level accuracy (i.e. in how far the specific items related to the singular competencies are capable of classifying applicants into categories of either possessing a specific competency or not) tells a different story: the results were acceptable for the V-SJT (Ethical awareness, 0.87; Empathy, 0.86; Reflection, 0.90; Medical and Societal Consciousness, 0.84; Collaboration, 0.99) and also for the WAT (Ethical awareness, 0.67; Reflection, 0.67; Medical and Societal Consciousness, 0.67; Transfer, 0.89; Textual Comprehension and Reasoning, 0.78; Organization, 0.82). As stated before, the V-SJT focuses on the more (inter)personal competencies in the blueprint, while the WAT focuses on a broader array of competencies, including the more cognitively loaded competencies. It is likely that this broadness of the WAT caused the low test level accuracy: applicants scoring high on the (inter)personal competencies may have scored low on the more cognitively-loaded ones, or the other way around, while others may have scored high or low on both, diminishing the overall test accuracy.

In the first year the current selection procedure was executed (2011), both the inter- and intra-rater reliability were determined and appeared to be > 0.95. Given the low interrater variability, this was not formally assessed in later years. To ensure reliability, intra-rater reliability was assessed across all five subsequent years and has been consistently ≥ 0.98.

Table 2 shows the item functioning results per test. While the applicants’ performance on the individual items differed somewhat, scores on the V-SJT items seemed to support the students’ suggestion that there was a time pressure effect, especially in the last two items. In the WAT (where applicants could browse through the test), this effect was less obvious. To determine whether there was a real time effect, an Omega analysis was conducted (solely for this purpose) for both tests. A time pressure effect was found for the last three assignments in the V-SJT and for the last two assignments in the WAT. Furthermore, the G-DINA found acceptable accuracies for time pressure (0.99 and 0.69 for the V-SJT and WAT, respectively). Therefore, time pressure was included as a construct in later analyses.

**Table 2:** Item functioning statistics for the applicant group as a whole (n = 547) and differential item functioning assessed for gender, pu-GPA and age

	Mean score% (SD)	Guessing parameter^a^	Slipping parameter^b^	Item-total correlation	Gender^c^ t (*p* value)	pu-GPA^d^ F (*p* value)	Age^e^ F (*p* value)
V-SJT							
1	64.06 (17.80)	0.30	0.99	0.04	2.02 (0.04)*	2.26 (0.13)	0.00 (0.98)
2	62.01 (20.46)	0.28	0.22	0.22	2.66 (0.01)**	2.11 (0.15)	0.19 (0.66)
3	60.46 (14.52)	0.19	0.00	0.21	− 0.68 (0.50)	4.24 (0.04)*	2.41 (0.12)
4	72.30 (19.80)	0.44	0.00	0.17	1.35 (0.18)	3.03 (0.08)	0.79 (0.38)
5	68.34 (20.68)	0.00	0.00	0.15	1.96 (0.05)	1.35 (0.25)	0.59 (0.44)
6	75.88 (18.30)	0.00	0.18	0.23	1.14 (0.25)	10.09 (0.00)**	7.52 (0.01)**
7	41.97 (19.46)	0.05	0.00	0.36	1.46 (0.15)	0.05 (0.83)	1.32 (0.25)
8	41.43 (23.48)	0.03	0.00	0.44	1.01 (0.32)	0.11 (0.74)	0.54 (0.46)
9	42.48 (30.91)	0.00	0.03	0.59	0.16 (0.87)	0.78 (0.38)	0.02 (0.90)
10	29.43 (29.85)	0.05	0.00	0.43	0.98 (0.33)	0.55 (0.46)	0.41 (0.52)
11	14.88 (23.08)	0.01	0.00	0.45	0.72 (0.47)	0.43 (0.52)	0.38 (0.54)
Written Aptitude Test							
1	48.74 (21.23)	0.38	0.44	0.21	− 2.81 (0.01)**	1.34 (0.25)	1.05 (0.31)
2	44.66 (13.77)	0.42	0.36	0.20	− 0.33 (0.74)	0.93 (0.34)	10.54 (0.00)**
3	61.29 (15.42)	0.01	0.04	0.21	1.01 (0.31)	0.95 (0.33)	0.43 (0.51)
4	35.66 (30.07)	0.07	0.12	0.25	− 3.05 (0.00)**	3.97 (0.05)*	0.36 (0.55)
5	40.35 (30.85)	0.26	0.55	0.17	− 1.29 (0.20)	2.31 (0.13)	0.42 (0.52)
6	43.48 (16.44)	0.20	0.38	0.20	0.62 (0.54)	5.63 (0.02)*	2.60 (0.11)
7	41.97 (19.46)	0.67	0.00	0.25	0.06 (0.95)	11.04 (0.00)**	0.28 (0.60)
8	41.43 (23.48)	0.02	0.01	0.21	0.78 (0.44)	5.64 (0.02)*	0.74 (0.39)

*Significant at *p *< 0.05

**Significant at *p *< 0.01

^a^Guessing is the probability that a respondent responds correctly to the item although he or she has not mastered all the required attributes; analyzed using the G-DINA model with 0 is low and 1 is high

^b^Slipping is the probability that a respondent responds incorrectly to the item although he or she has mastered all required attributes; analyzed using the G-DINA model with 0 is low and 1 is high

^c^Independent samples *t*-test with 0 = female, 1 = male; positive *t*-values represent higher mean scores for women than for men, negative *t*-values represent higher mean scores for men than for women

^d^Linear regression analysis with pu-GPA as independent variable and performance on each item as dependent variable. All significant results for pu-GPA are in favor of higher pu-GPAs

^e^Linear regression analysis with Age as independent variable and performance on each item as dependent variable; for item 6 on the V-SJT the older students had an advantage, while they had a disadvantage on item 2 of the written test

Applicants’ chances of getting items right through *guessing* were mostly low (under 0.5). Also, the chance an applicant possesses the competencies that are measured in an item but still got it wrong (i.e. *slipping*) were mostly low, except for the first item in the V-SJT and the fifth one in the WAT. For all items but the first V-SJT item, the item-total correlations were acceptable.

Table 2 also shows the results of the DIF analyses: the only factor increasing overall performance in the selection procedure was pu-GPA. Gender and age did not affect the overall performance throughout the selection procedure, as their effects outweigh themselves (two items in favor of men, two in favor of women; one in favor of older applicants, one in favor of younger applicants). The effect of pu-GPA was positive for six of the 19 items in the selection procedure. Mostly, these were items with high cognitive load (e.g. finding appropriate responses and ordering them or combining multiple bits of information to get to the correct answer), or items closely resembling high school content (e.g. textual comprehension or mathematical questions).

Finally, to gather evidence for the validity of the tools within the selection procedure (i.e. V-SJT and WAT) based on their internal structure, a saturated G-DINA model was applied (Sorrel et al. 2016). The test statistic used to determine which specific model was applied per attribute was based on the Wald test, the decision rule being “simpler model + largest *p* value rule at 0.05 alpha level; adjusted p values were based on Bonferroni correction” (Ma and De La Torre 2017). The results of the Q-matrix validation by G-DINA are shown in Table 3 (Time pressure was added to the blueprint; see above). It shows which competencies were expected and measured in which items of each test; Collaboration and Empathy were only assessed in the V-SJT, while Transfer, Textual comprehension, Reasoning, and Organization were only assessed in the WAT. The data consisted of only zeroes and ones, with zeroes meaning that a competency is not expected and measured in that item and a one that a competency is expected and measured in that item.

**Table 3:** Results of the Q-matrix validation as performed by G-DINA, with 0 meaning that this competency was not expected/measured by an item, and 1 meaning that this competency was expected/measured by an item

	Transfer	Text. and Reasoning	Collaboration	Organization	MSC	Ethical awareness	Empathy	Reflection	Time pressure
V-SJT									
1	NA	NA	0	NA	1	1	1	1	0
2	NA	NA	*0 → 1*	NA	0	*0 → 1*	1	1	*0 → 1*
3	NA	NA	0	NA	1	1	0	1	0
4	NA	NA	0	NA	1	1	1	1	0
5	NA	NA	1	NA	0	0	0	*1 → 0*	0
6	NA	NA	0	NA	1	1	1	1	0
7	NA	NA	1	NA	1	1	0	1	0
8	NA	NA	0	NA	1	1	1	1	0
9	NA	NA	0	NA	1	0	1	1	1
10	NA	NA	1	NA	0	0	*1 → 0*	1	1
11	NA	NA	0	NA	1	1	0	0	1
WAT									
1	1	*0 → 1*	NA	0	*0 →* 1	0 → 1	NA	*0 → 1*	0
2	1	1	NA	*0 → 1*	0	*0 → 1*	NA	*0 → 1*	0
3	0	0	NA	0	1	1	NA	1	0
4	1	0	NA	0	0	0	NA	0	0
5	*0 → 1*	1	NA	*0 → 1*	0	0	NA	0	0
6	0	1	NA	0	0	0	NA	0	0
7	0	0	NA	0	1	1	NA	1	1
8	0	0	NA	1	0	0	NA	0	1

Italics and two numbers with an arrow between them indicate that the Q-matrix was changed during the Q-matrix validation analysis; the first number is from the Q-matrix based on the blueprint (expected), the second number is the result of the G-DINA analysis and due to the applicants’ scores (measured). All other numbers were expected and measured

*NA* not applicable; *V-SJT* video-based situational judgement test; *WAT* written aptitude test; *Text. & reasoning* textual comprehension and reasoning; *MSC* medical and societal consciousness

The AIC and BIC model fits were calculated for each test. For the V-SJT, they were 7818.46 and 8675.05, respectively, and for the WAT they were 6198.89 and 6917.73, respectively. By applying the changes to the Q-matrices suggested by the G-DINA, the model fit does not increase significantly; therefore, the original Q-matrix and suggested changes are provided. A G-DINA analysis of both tests together would result in a drastic decrease of the model fit (AIC = 14,166.52 and BIC = 17,123.67), which is logical as they are simply different tests. Because of these reasons, the V-SJT and WAT were analyzed separately.

As shown in Table 3, SME predictions were overruled by the G-DINA analysis in only 14 of the 122 cases (i.e. 14 of the 122 predictions of which derived competencies were and were not measured by which items were incorrect according to the G-DINA analysis); this is illustrated by the fact that two numbers are shown with an arrow between them. In these cases, items measured other and/or additional competencies than expected by the SMEs. For example, the fifth item in the V-SJT was in fact not measuring Reflection, but did measure Collaboration, the other competency that was intended to be measured. The change was the other way around for the fifth item in the WAT; this item was shown to not only measure Textual comprehension and reasoning, but also Transfer and Organization. All in all, these results show that there is an overlap between expected and measured competencies of 92% for the V-SJT and of 84% for the WAT, adding up to an overlap of 89% between the predetermined, expected Q-matrix for the overall selection procedure and the matrix as validated using G-DINA. Furthermore, the majority of changes that the analysis made to the expected Q-matrix were explicable when the results per item were investigated further and cross-checked with an SME.

## Discussion

The aim of this study was to investigate the evidence related to the construct validity of our selection procedure, in order to open the ‘black box of selection’. Our specific focus was on content and internal structure, as these shed the most light into this black box. The set-up of the selection procedure proved to be a multi-step and robust process to determine content, which was transparent and replicable, and translated into representative items according to the applicants. Moreover, the G-DINA Q-matrix validation indicated 89% overlap between the expected and actually measured competencies for the items of the V-SJT and WAT. This shows that focusing on the right content by following the competency blueprint was effective in terms of internal structure, and that we are really measuring what we want to measure.

The majority of the evidence presented in the current study is supportive of the selection procedure’s construct validity. Related to the content of the selection procedure, we found that it was possible for a group of committed SMEs to form a selection committee proficient in carefully creating a blueprint of derived competencies needed for medical school and constructing tests capable of distinguishing between applicants based on these competencies. The applicants agreed with the idea that the selection procedure was fairly representative; they indicated that they could accurately portray their abilities in the selection tests and that the selection procedure as a whole contained all aspects needed to identify suitable candidates. All in all, the process of gathering content for the selection procedure appears to be robust, transparent and replicable. Moreover, from previous research we already know that the current procedure is predictive for pre-clinical (Schreurs et al. 2018) and clinical (Schreurs et al. 2019) performance during medical school.

Related to the internal structure of both tests, there seems to be a huge overlap (89%) between the expected and actually measured competencies in both the V-SJT and the WAT. This indicates that the internal structure of the tests used in the selection procedure mostly reflects the content that was intended to be measured. Although the overall test accuracy was only acceptable for the V-SJT, both tests showed acceptable attribute level accuracies (≥ 0.84 for the V-SJT and ≥ 0.67 for the WAT). As stated before, the difference in test accuracy between both tests is likely caused by the fact that the WAT measured a broader range of competencies (i.e. both (inter)personal and cognitively-loaded ones), while the V-SJT focused specifically on the more (inter)personal competencies. Importantly, the inter-rater as well as the intra-rater reliabilities were very high. In both tests, time pressure was found to influence the applicants’ performance in the last few items, which was in line with the applicants’ comments. As a result, time pressure was included as a construct in the G-DINA analysis, which confirmed its effect.

Taking time pressure into account, we looked at several other effects. Firstly, the guessing parameter (i.e. the probability that a respondent responds correctly to the item although, based on the scores of the other items, he or she has not mastered all the required attributes) was low (< 0.5) for most items. The only item with a higher chance of getting it right through guessing appeared to be item 7 in the WAT. The topic presented in this item was relevant but the text was formulated in a complex manner, which may have introduced a high cognitive load. The latter is supported by the highly significant DIF and relatively large effect size of pu-GPA for this particular item. The relatively high guessing parameter for this item may therefore indicate that performance on this item is related to the applicants’ pu-GPA rather than their competencies. The slipping parameter (i.e. the probability that a respondent responds incorrectly to the item although, based on the scores of the other items, he or she has mastered all required attributes) was found to be low for most items as well. The only item with a very high slipping parameter was the first item in the V-SJT. This may mean that this item is actually not measuring what was intended to be measured, which is supported by this item’s low item-total correlation. This may be due to the ‘first-item effect’, caused by the fact that the V-SJT is a new kind of test to most applicants, that they have to make under high pressure with a lot at stake. This suggests that each first item would suffer from this effect. Further examination in later years has to demonstrate whether this explanation is valid. The only other item with a relatively high slipping parameter was the fifth item in the WAT. This item was a specific test of fluid intelligence [i.e. “defined as reasoning ability, and the ability to generate, transform, and manipulate different types of novel information in real time” (Zaval et al. 2015)]. However, in hindsight, the competency that was intended to be measured (Textual comprehension and reasoning) was too broad for this specific item.

As for the Differential Item Functioning, age and gender did not affect overall performance: some of the items showed some effects of age and gender, but they outweighed themselves. Pu-GPA, however, was significantly and positively related to performance on two of the V-SJT items and four of the WAT items. Interestingly, the V-SJT scores were less affected by pu-GPA, which probably relates to the fact that the V-SJT primarily assessed the more (inter)personal competencies.

The most important analysis applied in answering the question whether effortful creation of the content of a selection procedure, based on a blueprint, leads to an internal structure in line with that blueprint was the G-DINA. The G-DINA results show that the large majority of the expected competences as reflected in the blueprint were actually measured in both the V-SJT (92% overlap) and the WAT (84% overlap). The changes proposed by the G-DINA were critically assessed by the authors, and in hindsight, most changes make sense, while some do not. These results warrant further investigation.

Some novelties in the current study are worth highlighting. First, the application of Cognitive Diagnostic Modeling (CDM). Although García et al. (2014) already applied CDM to an SJT used for selection in the financial sector in 2014, its application in medical education research is new. Like García et al., we conclude that this emerging analytical method is appropriate for SJT data as well as for selection data in general; it easily copes with multidimensionality, not only between but also within the items. Furthermore, CDM fits the purpose of the current study perfectly; it indicates whether the items were measuring what they were intended to measure, and whether other competencies unintendedly were measured as well. As a consequence, it is possible to investigate the construct validity of selection processes in addition to their predictive and incremental value. Therefore, applying CDM is the main implication of the current study: it is an extremely versatile test theory that is highly applicable to selection procedures. Importantly, it is easily integrated into validity arguments according to modern validity theories (e.g. AERA, APA and NCME 2014; Downing 2003). Therefore, these analyses can be applied at other educational institutes as well, to help them understand their selection procedures more thoroughly and to gather information on the validity of their procedures.

The second important novelty is that, to the best of our knowledge, this is the first time Downing’s validity framework has been used to assess evidence related to the construct validity of an outcome-based selection procedure. In the current study, we chose to focus on only two aspects of construct validity in Downing’s framework (2003): *content* and *internal structure*. Our previous research provides information on two other aspects of the framework. With regard to *relation to other variables*, a positive relation has been shown between being selected and study success throughout the medical (pre-clinical) bachelor (Schreurs et al. 2018) and clinical master (Schreurs et al. 2019). Related to the *consequences* of the selection procedure, the cost-effectiveness of the MUMS selection procedure was investigated as compared with a lottery procedure, and it was found that even though selection requires a significant financial investment, the benefits in the medical bachelor already outweigh the costs of the whole procedure (Schreurs et al. 2018). For evidence related to *response processes*, no thorough empirical research has been performed yet. This is an important future direction for research and a limitation for the current study.

Our findings illustrate that research on selection for medical school can focus on more than predictive validity alone. Investigating construct validity with the help of validity frameworks offers a more general evidence base for the application of selection procedures, making them more defensible and fair. Furthermore, applying newer test theories such as CDM provides information on which constructs are indeed measuring what they were intended to measure, and which should be excluded. In the current study, we have shown that the use of CDM can offer new ways to ameliorate selection procedures. It enables a critical reflection on the value of individual tools and items, and opens ways to make these high-stakes procedures more justifiable and fair. On the basis of CDM, the local selection committee has grown more critical towards the competencies intended to be measured per item.

A limitation of this study was the need to dichotomize the responses of the applicants to enable their use in the G-DINA rather than using a polytomous approach, because of the relatively small sample size. Due to the necessary dichotomization of the data, some of the richness of the data was lost for the G-DINA. Nevertheless, the current results show a huge and convincing overlap with the original blueprint, supporting the construct validity of our selection procedure. Furthermore, all other analyses were conducted using the raw data. The current use of G-DINA can be considered as an initial exploration of its potential in the analysis of medical school selection. More studies applying CDM to selection (and other areas of Health Professions Education) are highly welcome, as are comparisons between dichotomized and polytomous CDM analyses. Another limitation of this study is the use of just one cohort from one institution. Further studies in other contexts are necessary to investigate whether the results obtained in the present investigation are generalizable. Also, the current study focused on the second round of the selection procedure alone, and validity evidence should be gathered for the procedure as a whole. Therefore, in future studies, the entire selection procedure should be taken into account. An important gap to fill in the general selection literature is also the issue of weighting: how should different constructs and/or tools be weighted in order to achieve the most valid selection procedure?

We conclude that a carefully built blueprint is not only useful to obtain a good level of content validity for medical school selection, but it also proved to have an important positive impact on the quality of the results in terms of internal structure. By linking the blueprint to the applicants’ results, we established that we are indeed measuring the constructs we intended to measure, therewith shedding light in the ‘black box of selection’. We believe this study shows that it is possible to evaluate the construct validity of medical school selection.
